# Peak atrial longitudinal strain as a predictor of postoperative atrial fibrillation in isolated coronary artery bypass grafting: Insights from a single-centre study in Indonesia

**DOI:** 10.21542/gcsp.2025.58

**Published:** 2025-10-31

**Authors:** Ahmad F. Lubis, Andre P. Ketaren, Abdul H. Raynaldo, Anggia C. Lubis, Teuku B. Haykal, Tengku W. Ardini, Joy W. Purba, Nizam Z. Akbar

**Affiliations:** 1 Cardiovascular Department, Adam Malik General Hospital, Medan, 20136, Indonesia; 2 Department of Cardiology and Vascular Medicine Faculty of Medicine, Universitas Sumatera Utara, Medan, 20222, Indonesia; 3 Cardiovascular Department, Haji General Hospital, Medan, 20371, Indonesia

## Abstract

Background: Postoperative atrial fibrillation (POAF) is a frequent complication in patients undergoing coronary artery bypass grafting (CABG) and contributes to increased morbidity and mortality. Identifying accurate preoperative predictors for POAF remains a clinical challenge.

Objectives: This study aimed to evaluate the predictive value of preoperative peak atrial longitudinal strain (PALS) for POAF in patients undergoing isolated CABG.

Methods: This prospective cohort study was conducted from May to October 2024. A total of 58 adult patients undergoing isolated CABG were consecutively enrolled. PALS measurement was performed preoperatively using speckle tracking echocardiography. Patients were followed for 30 days postoperatively to monitor POAF occurrence. Statistical analyses in this study included ROC curve, Kaplan–Meier, and Cox regression.

Results: Of the 58 patients, 30 patients had PALS <28% and 28 patients had PALS ≥28%. The incidence of POAF was significantly higher in the low PALS group (53.3% vs 10.7%, *p* = 0.001). Multivariate analysis showed that low PALS, high BMI, and on-pump CABG were independent predictors of POAF. We also revealed that the combination of low PALS and on-pump CABG technique conferred the highest risk of POAF. Kaplan–Meier analysis reinforced that the cumulative incidence of POAF was higher in patients with low PALS and those undergoing on-pump CABG.

Conclusion: Preoperative PALS < 28% is a strong predictor of POAF in patients undergoing isolated CABG. PALS assessment and surgical technique can optimize risk stratification and POAF prevention strategies.

## Introduction

Postoperative atrial fibrillation (POAF) is one of the most common and challenging complications in patients undergoing cardiac surgery, especially coronary artery bypass grafting (CABG)^1^. The incidence of POAF in CABG patients ranges from 20% to 40%, contributing to increased morbidity, prolonged hospital stays, and mortality^2^. The occurrence of POAF is influenced by various factors such as advanced age, hypertension, heart failure, left ventricular dysfunction, and left atrial enlargement^3^. One factor that has gained particular attention is left atrial dysfunction, which can be measured non-invasively and is closely related to the pathogenesis of POAF^4^. Recent studies have shown that impaired left atrial mechanical function, especially related to decreased reservoir and contractile capacity, is a key mechanism underlying postoperative arrhythmias, making the assessment of left atrial function increasingly important in POAF risk stratification^4,5^.

Peak atrial longitudinal strain (PALS) is an echocardiographic parameter used to quantitatively assess left atrial mechanical function through speckle tracking echocardiography (STE)^6^. PALS reflects the elasticity and stretching ability of the left atrium during the reservoir phase, which is strongly influenced by filling pressures and atrial tissue structure^7^. Decreased PALS values have been associated with increased risk of POAF in several studies, as they indicate fibrosis, remodeling, or elevated left atrial pressure^8^. However, some studies have reported inconsistent results, and to date, no universal PALS cutoff value has been widely adopted in clinical practice.^9^ Furthermore, the impact of surgical technique (on-pump versus off-pump CABG) on the relationship between PALS and POAF remains debated^10,11^. These issues highlight the need for further research to specifically evaluate the relationship between preoperative PALS values and POAF occurrence in isolated CABG patients, as well as to assess the influence of surgical technique on this relationship.

This study aims to evaluate the predictive ability of preoperative PALS values for POAF in patients undergoing isolated CABG. We hypothesize that low PALS value significantly increases the risk of POAF, especially in patients undergoing on-pump procedures. If confirmed, PALS assessment could be integrated as a POAF risk screening tool, enabling more targeted prevention and management strategies in the CABG patient population^12^.

## Methods

### Design

This study was a prospective cohort conducted at Adam Malik General Hospital, Medan, Indonesia, with data collection from May to October 2024. The approach involved recruiting patients who met the inclusion and exclusion criteria for isolated CABG surgery. All participants underwent left atrial strain analysis before surgery and were followed for 30 days postoperatively to assess the occurrence of POAF.

### Participants & eligibility criteria

**Figure 1.**
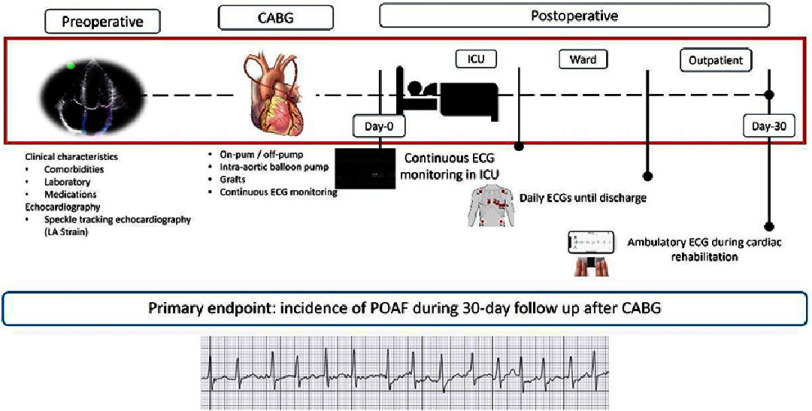
Study illustration.

The sample consisted of 58 adult patients scheduled for isolated CABG, selected by consecutive sampling. To ensure that our sample size was adequate to detect clinically meaningful differences between two groups, we performed post-hoc power analysis using G*Power. Inclusion criteria were adult patients planned for isolated CABG with complete clinical, echocardiographic, and laboratory data. Exclusion criteria included patients undergoing concomitant valve intervention, history of maze procedure or left atrial appendage exclusion, acute myocardial infarction within 30 days before CABG, previous atrial fibrillation, clinical or subclinical hypothyroidism, poor echocardiographic window, use of antiarrhythmic drugs, and patients who died before POAF occurrence or before completing the 30-day follow-up (Figure 1).

### Ethical approval

This study received ethical approval from the Health Research Ethics Committee of Universitas Sumatera Utara (1027/KEPK/USU/2024). All procedures adhered to the principles of the Declaration of Helsinki, and all participants provided written informed consent after receiving a full explanation of the study’s purpose, benefits, and risks. Patients had the right to withdraw from the study at any time. No incentives were provided to the patients.

### Data collection

In detail, patient underwent a thorough and extensive clinical evaluation, where medical history, cardiovascular risk factor, comorbidities and medications were recorded. Blood samples also obtained before surgery. Baseline echocardiographic examination were performed 24 h before the surgery, which then completed offline by speckle tracking echocardiography (STE) analysis. To assess measurement variability, intra-observer and inter-observer variability were evaluated in a randomly selected subset of 15 patients. The same observer repeated measurements one month apart to determine intra-observer variability, while a second observer analyzed these data to obtain inter-observer variability. Variability was quantified using interclass correlation coefficients (ICC). Operative details related to on-pump/off-pump surgery, numbers of graft, and the use of intra-aortic balloon pump (IABP) machine were also recorded. After the operation, patients were followed in the intensive care unit for at least 48 h and then in the cardiac surgery ward.

During intensive care unit stay, all patients underwent continuous 12-lead ECG monitoring. Then, during the hospital stay in the ward, cardiac rhythm was assessed with daily ECGs until discharge and ECG was also done in the case of suggestive symptoms. After the discharged, all patients underwent cardiac rehabilitation for a month with 12 visits. During this period of time, the ECGs were obtained before starting the exercise by using single-lead ECG device which connected to the smartphone. This is a portable device that records a single electrical tracing of the heart by using finger as one of the electrodes. AF diagnosis was based on the detection of a standard 12-lead ECG recording or a single-lead ECG tracing of irregular rhythm with no discernible repeating P waves (irregular RR intervals) in absence of impaired atrioventricular conduction, of ≥30 s duration and/or requiring pharmacological cardioversion. In patients with >1 POAF event, the time of the first event was used for the analysis (Supplementary Figure 1).

### Covariates

The main variable analyzed was PALS, defined as the maximum positive strain value of the left atrium measured by speckle tracking echocardiography in the apical four-chamber view according to ASE guidelines. PALS measurement was performed by tracing the endocardial and epicardial borders of the left atrium, excluding the pulmonary veins and left atrial appendage, with a region of interest thickness of three mm. The R wave on ECG was used as the starting point for strain calculation, producing reservoir, conduit, and contraction strain values.

We determined the cut-off value of PALS <28% and ≥28% based on the previous cohort prospective and multicentre study to divide our samples into two groups. PALS <28% was considered low and associated with a higher risk of POAF^13^. Other variables analyzed included age, sex, comorbidities, medication use, echocardiographic parameters (EF, TAPSE, LAVi, E/A, E/e’), laboratory values (blood count, creatinine, creatinine clearance, electrolytes), perioperative factors (use of IABP, on-pump CPB, number of grafts), and postoperative outcomes (POAF, length of hospital stay, mortality).

### Statistical analysis

Categorical data were presented as *n* (%). The Kolmogorov–Smirnov test was used to assess the normality of numerical data. Normally distributed data were presented as mean ±  standard deviation (SD); non-normally distributed data as median (interquartile range, IQR). Baseline comparisons between groups were performed using the independent *t*-test for normally distributed numerical variables, Mann–Whitney test for non-normally distributed numerical variables, and chi-square or Fisher’s exact test for categorical data. Receiver operating characteristic (ROC) curve analysis was used to evaluate the predictive value of PALS for POAF. Bivariate analysis was conducted using chi-square or Fisher’s exact test for categorical variables and independent *t*-test or Mann–Whitney test for numerical variables, as appropriate. Kaplan–Meier curves and log-rank tests were used to assess POAF-free survival up to 30 days. Cox proportional hazards regression was performed to identify independent predictors of POAF, reporting hazard ratios (HR) and 95% confidence intervals (CI). Variables included in multivariate analysis were also tested for collinearity by linear regression analysis. All analyses were performed using SPSS for Windows, with *p* values <0.05 considered statistically significant.

## Results

### Patient selection flowchart

**Figure 2.**
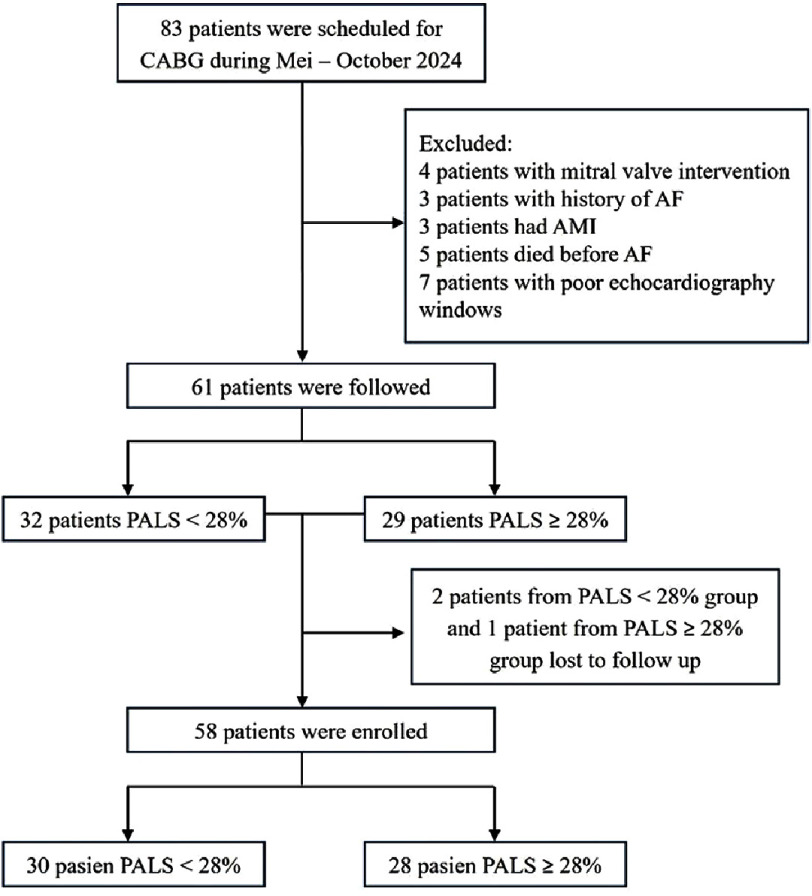
A flowchart of patient selection in our study. Notes, AF, atrial fibrillation; AMI, acute myocardial infarction; CABG, coronary artery bypass graft; PALS, peak atrial longitudinal strain.

The patient selection process is illustrated in Figure 2, which depicts the screening flow from the initial population to the final 58 patients who met the inclusion criteria. Patients with a history of atrial fibrillation and acute myocardial infarction were excluded from the analysis. The final group was then classified based on preoperative PALS values into two main groups for further comparative analysis. This flow structure provides clarity regarding the distribution of case and control samples in the study.

**Table 1:** Baseline characteristics.

Variable	Total sample (*n* = 58)	PALS < 28% (*n* = 30)	PALS ≥28% (*n* = 28)	*P* value
Preoperative parameters				
Male sex, *n* (%)	53 (91.4)	28 (93.3)	25 (89.3)	0.665
Age, years	58.6 ± 5.8	57.8 ± 5.8	59.4 ± 5.8	0.311
Age ≥ 60 years, *n* (%)	23 (39.7)	11 (36.7)	12 (42.9)	0.831
BMI, kg/m^2^	25.6 ± 3.6	25.9 ± 3.9	25.6 ± 3.2	0.809
BMI ≥ 25 kg/m^2^, *n* (%)	32 (55.2)	18 (60)	14 (50)	0.616
Diabetes melitus, *n* (%)	20 (34.5)	12 (40)	8 (28.6)	0.523
Hypertension, *n* (%)	27 (46.6)	17 (56.7)	10 (35.7)	0.182
Smoking, *n* (%)	33 (56.9)	16 (53.3)	17 (60.7)	0.763
Chronic heart failure, *n* (%)	29 (50)	20 (66.7)	9 (32.1)	0.018
Beta bloker, *n* (%)	53 (91.4)	27 (90)	26 (92.9)	1.000
ACEi/ARB, *n* (%)	46 (79.3)	26 (86.7)	20 (71.4)	0.268
CCB dyhidroperidin, *n* (%)	15 (25.9)	7 (23.3)	8 (28.6)	0.877
Echocardipgraphy parameters				
EF, %	54.5 (15–70)	50 (15–65)	58 (20–70)	0.014
EF ≤ 40%, *n* (%)	19 (32.8)	13 (43.3)	6 (21.4)	0.135
TAPSE, mm	20 (9–29)	19 (9–29)	21 (13–27)	0.003
Left atrial strain, %				
Reservoir strain	26.6 (7.03–42)	N/A	N/A	N/A
Conduit strain	12.7 (2.19–27.9)	N/A	N/A	N/A
*Contraction strain*	11.8 (2.03–23.3)	N/A	N/A	N/A
LAVi, mL/m^2^	24.85 (11.2–55.67)	25.1 (11.2–55.7)	23.6 (12.7–50.9)	0.641
LAVi > 34 mL/m^2^, *n* (%)	17 (29.3)	11 (36.7)	6 (21.4)	0.324
E/A	0.87 (0.38–1.8)	1.1 ± 0.5	0.8 ± 0.2	0.007
E/e’	9.63 ± 2.00	9.9 ± 2.08	9.25 ± 1.88	0.163
Laboratory parameters				
Haemoglobin, g/dL	13.7 (8.2–16.5)	12.9 ± 1.7	13.7 ± 1.9	0.123
WBC, 10^3^/μL	8.9 (4.7–18.2)	9.3 ± 2.9	9.2 ± 2.5	0.845
Platelet, 10^3^/μL	248 (137–437)	267 ± 74.8	263 ± 65.5	0.824
Creatinin, mg/dL	1.16 (0.58–2.8)	1.2 (0.7–2.8)	1.07 (0.58–2.17)	0.188
Creatinine clearance,	62.5 (30–137)	59.5 (30–137)	66 (30–121)	0.379
Magnesium	2.5 (1.36–4.82)	2.3 (1.36–4.82)	2.5 (1.51–3.91)	0.803
Kalium	3.7 (2.9–6.1)	3.6 (3–6.1)	3.7 (2.9–4.5)	0.888
Calcium	8,62 ± 0,61	8,59 ± 0,65	8.65 ± 0,56	0,730
Perioperative parameters				
IABP, *n* (%)	6 (10.3)	5 (16.7)	1 (3.6)	0.195
CPB machine, *n* (%)				
On-pump, *n* (%)	26 (44.8)	16 (53.3)	10 (35.7)	0.278
Off-pump, *n* (%)	32 (55.2)	14 (46.7)	18 (64.3)	0.278
Graft numbers	3 (2–5)	3 (2–5)	3 (2–4)	0.098
Postoperative parameters				
POAF, *n* (%)	19 (32.8)	16 (53.3)	3 (10.7)	0.001
Length of stay, days	9 (2–28)	9.5 (2–28)	8.5 (2–20)	0.154

**Notes.**

BMI body mass index EF ejection fraction TAPSE tricuspid annular plane systolic excursion LAVi left atrial volume index E/A ratio of early (E) to late (A) ventricular filling velocities E/e’ ratio of early mitral inflow velocity to mitral annular early diastolic velocity WBC white blood cell count IABP intra-aortic balloon pump CPB cardiopulmonary bypass POAF postoperative atrial fibrillation ACEi/ARB angiotensin-converting enzyme inhibitor/angiotensin receptor blocker CCB dyhidroperidin dihydropyridine calcium channel blocker N/A not available

**Table 2:** Bivariate analysis based on the presence/absence of POAF.

Variable	POAF *n* = 19	No POAF *n* = 39	*P* value
Male sex, *n* (%)	17 (89.5)	36 (92.3)	1.000
Age, years	59.26 ± 4.58	58.26 ± 6.35	0.541
Age ≥ 60 years, *n* (%)	10 (52.6)	13 (33.3)	0.261
BMI, kg/m^2^	26.4 (19.3–37.3)	24.6 (18.67–32.34)	0.036
BMI ≥ 25 kg/m^2^ , *n* (%)	16 (84.2)	16 (41)	0.005
Diabetes melitus, *n* (%)	9 (47.4)	11 (28.2)	0.251
Hypertension, *n* (%)	13 (68.4)	14 (35.9)	0.04
Smoking, *n* (%)	11 (57.9)	22 (56.4)	1.000
Chronic heart failure, *n* (%)	13 (68.4)	16 (41)	0.093
Beta bloker, *n* (%)	16 (84.2)	37 (94.9)	0.390
ACEi/ARB, *n* (%)	17 (89.5)	29 (74.4)	0.323
CCB dyhidroperidin, *n* (%)	7 (36.8)	8 (20.5)	0.311
EF, %	53 (15–66)	55 (18–70)	0.697
EF ≤ 40%, *n* (%)	8 (42.1)	11 (28.2)	0.447
TAPSE, mm	19 (9–29)	21 (13–28)	0.013
LAVi, mL/m^2^	27.8 ± 12.84	27.8 ± 10.24	0.999
LAVi > 34 mL/m^2^, *n* (%)	16 (84.2)	14 (35.9)	0.001
E/A	0.8 (0.38–1.4)	0.89 (0.4–2.8)	0.914
E/e’	9.06 ± 2.15	9.9 ± 1.89	0.531
Haemoglobin, g/dL	12.92 ± 1.97	13.5 ± 1.78	0.296
WBC, 10^3^/μL	9.2 ± 2.5	9.3 ± 2.8	0.858
Platelet, 10^3^/μL	262 ± 75.1	267 ± 68.1	0.787
Creatinin, mg/dL	1.3 (0.74–1.88)	1.1 (0.58–2.8)	0.059
Creatinine clearance	56 (39–137)	66 (30–121)	0.233
Magnesium	2.78 (1.63–4.82)	2.45 (1.36–4.1)	0.456
Kalium	3.9 (3.1–6.1)	3.7 (2.9–5.3)	0.141
Calcium	8.45 ± 0.58	8.7 ± 0.6	0.443
IABP, *n* (%)	3 (15.8)	3 (7.7)	0.382
On-pump CPB, *n* (%)	15 (78.9)	11 (28.2)	0.001
Graft numbers	3 (2–4)	3 (2–5)	0.942
Length of stay, days	11 (2–28)	9 (6–20)	0.129
Mortality, *n* (%)	3 (15.8)	0 (0)	0.031

**Notes.**

BMI body mass index ACEi/ARB angiotensin-converting enzyme inhibitor/angiotensin receptor blocker CCB dyhidroperidin dihydropyridine calcium channel blocker EF ejection fraction TAPSE tricuspid annular plane systolic excursion LAVi left atrial volume index E/A ratio of early (E) to late (A) ventricular filling velocities E/e’ ratio of early mitral inflow velocity to mitral annular early diastolic velocity WBC white blood cell count IABP intra-aortic balloon pump CPB cardiopulmonary bypass POAF postoperative atrial fibrillation

### Baseline characteristics of patients included in our analysis

A total of 58 patients undergoing isolated CABG were analyzed, consisting of 30 patients with PALS <28% and 28 patients with PALS ≥28%. Of note, intra-observer and inter-observer variability showed ICC of 0.891 (95% CI: 0.77–0.92) and 0.952 (95% CI: 0.56–0.86), indicating a very high reliability measurement. As shown in Table 1, several variables demonstrated statistically significant differences between the two groups, including chronic heart failure (*p* = 0.018), ejection fraction (*p* = 0.014), TAPSE (*p* = 0.003), and E/A ratio (*p* = 0.007). Additionally, the incidence of POAF was significantly higher in the low PALS group (53.3% vs 10.7%, *p* = 0.001).

### Bivariate analysis based on the presence or absence of POAF

Analysis of 58 patients, including 19 with POAF and 39 without POAF, revealed that several clinical and perioperative variables differed significantly between the groups, as seen in Table 2. Significant *p*-values were found for BMI (*p* = 0.036), proportion of BMI ≥ 25 kg/m^2^ (*p* = 0.005), hypertension (*p* = 0.04), TAPSE (*p* = 0.013), LAVi > 34 mL/m^2^ (*p* = 0.001), and on-pump CABG surgical technique (*p* = 0.001). Comparative effects indicated that POAF was more frequent in patients with right ventricular dysfunction, left atrial enlargement, and more invasive surgical procedures. In-hospital mortality also occurred only in the POAF group (*p* = 0.031).

### Predictive value of PALS and multivariate analysis

**Figure 3.**
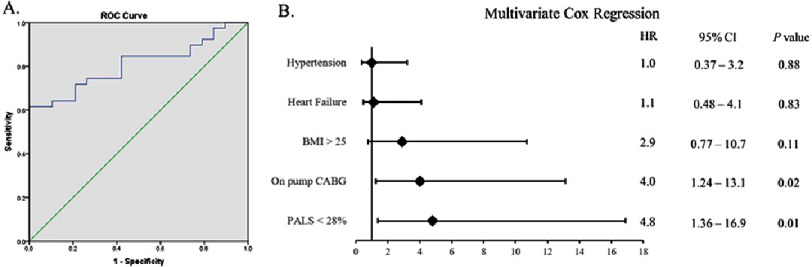
(A) Receiver operating characteristic curve showing the predictive value of peak atrial longitudinal strain (PALS) for postoperative atrial fibrillation (POAF). (B) Forest plot for multivariate analysis for predicting POAF. HR indicates hazard ratio; BMI, body mass index; PALS, peak atrial longitodinal strain.

Predictive analysis showed that PALS had good discriminative ability for POAF occurrence. Figure 3A presents the ROC curve with a high area under the curve for PALS as a predictor of POAF (AUC 0.807) with sensitivity of 84.2% and specificity of 64.1%,. The PALS <28% cut-off demonstrated good discrimination (C-statistic 0.783, 95% CI: 0.67–0.88) and adequate calibration (Hosmer-Lemeshow *p* = 0.416). At this threshold, positive predictive value (PPV) was 53.5% (95% CI: 41.85–64.47%) and negative predictive value (NPV) was 89.3% (95% CI: 74.18–96%). The number needed to screen was 2.3 patients (95% CI: 1.8–3.2), meaning approximately every 2–3 patients assessed with PALS could prevent on POAF event with appropriate intervention

Figure 3B displays a forest plot from multivariate analysis, indicating that PALS <28% and on-pump CABG use were independent predictors of POAF with significant *p*-values. Hazard ratio comparisons suggested that these two variables independently contributed to increased risk of postoperative atrial fibrillation. In addition, none of those varibles included in multivariate testing showed any multicollinearity (tolerance >0.1 and variance inflation factio (VIF) >10). Interaction testing by using linear regression showed that on-pump CABG had a stronger effect for the occurance of POAF compared to off-pump CABG (R^2^ 0.482; *p* = 0.001 and R^2^ 0.019; *p* = 0.458, respectively). Surgical technique showed an interaction with PALS value and could moderate the outcome of POAF (*F* = 10.97).

**Figure 4.**
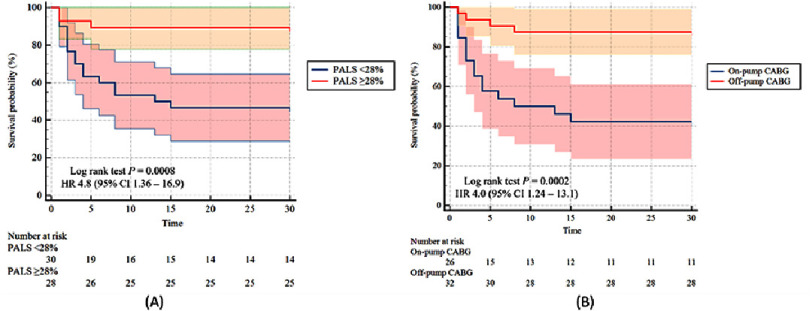
(A) Estimated rates of POAF by Kaplan–Meier method according to preoperative PALS value, (B) Estimated rates of POAF by Kaplan–Meier method according to on-pump CABG.

**Figure 5.**
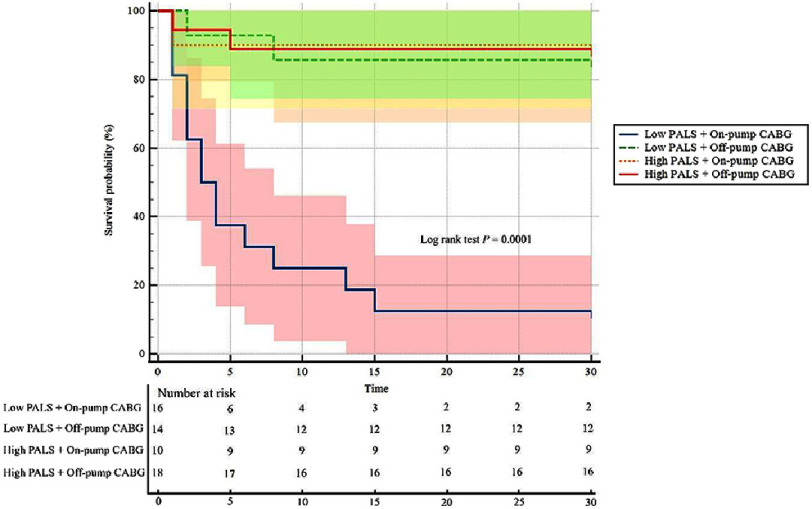
Estimated rates of POAF analyzed by Kaplan–Meier method according to different PALS values and surgical techniques.

### Kaplan–Meier POAF risk estimation based on PALS and CABG technique

The cumulative incidence of POAF estimated by Kaplan–Meier analysis in 58 patients is shown in Figure 4. The overall restricted mean survival time for POAF was 21.5 days (95% CI: 18.4–24.7). Figure 4A demonstrates a higher incidence of POAF in patients with PALS <28%. Patients with low PALS values significantly had worse survival time compared to group with higher PALS values (16.5 days, 95% CI: 11.8–21.15 vs. 27 days, 95% CI: 23.8–30.2, *p* = 0.001) and also 4.8 times more likely to develop POAF (95% CI: 1.36–16.9, *p* = 0.0008). Figure 4B shows that on-pump CABG technique increases the risk of POAF compared to off-pump. Patients with on-pump CABG significantly had worse survival time compared to group with off-pump CABG (15.2 days, 95% CI [10.2–20.2] vs. 26.7 days, 95% CI: 23.7–29.7, *p* = 0.001) and also 4 times more likely to develop POAF (95% CI: 1.24–13.1, *p* = 0.0002).

Figure 5 demonstrates a cumulative incidence of POAF estimated by Kaplan–Meier analysis across different PALS values combined with surgical technique. Patients with low PALS values combined with on-pump CABG showed the worst mean POAF-free time compared to other groups (7.8 days, 95% CI: 3.2–12.3, *p* = 0.0001), followed by low PALS with off-pump CABG (26.4 days, 95% CI: 21.8–31.1), both high PALS with on-pump CABG and high-PALS with off-pump CABG had the same mean POAF-free time with 27 days (95% CI: 21.7–32.5 and 95% CI: 23.1–30.9, respectively). Patients with low PALS combined with on-pump CABG also had a higher risk to develop POAF compared to whether the patients had low PALS only or underwent on-pump CABG only (HR 7.3, 95% CI: 3.1–17.1, *p* = 0.0001).

## Discussion

### Principal findings

In this study, 58 patients undergoing isolated CABG surgery were analyzed to assess the predictive ability of PALS for the occurrence of POAF. The main finding showed that PALS values <28% were significantly associated with an increased incidence of POAF, even after adjusting for other variables such as BMI, hypertension, and surgical technique^13^. Additionally, low PALS correlated with poorer cardiac function, including lower ejection fraction, decreased TAPSE, and left atrial enlargement^14^. Compared to previous studies, these results are consistent with findings that left atrial dysfunction detected non-invasively through strain analysis is a strong predictor of POAF^15^. Directly, low PALS reflects impaired left atrial mechanical function, making the atrium more susceptible to postoperative arrhythmias^14^. Indirectly, low PALS is often found in patients with other cardiovascular comorbidities, cumulatively increasing the risk of POAF^16,17^. Thus, our findings reinforce that preoperative PALS is an effective risk stratification tool to predict POAF in isolated CABG patients^13^.

Our results also found that on-pump CABG surgical technique, as well as the combination of low PALS and on-pump CABG, significantly increased the risk of POAF compared to other groups^10^. The highest incidence of POAF was observed in patients with PALS <28% undergoing on-pump surgery, while the lowest risk was in patients with PALS ≥28% undergoing off-pump surgery^18^. This finding aligns with several studies reporting that on-pump procedures may cause higher systemic inflammation and oxidative stress, worsening the arrhythmogenic substrate in the left atrium^2^. The combined effect of left atrial dysfunction (low PALS) and invasive surgical technique suggests a synergistic interaction that significantly elevates POAF risk^19^. Directly, this effect can be explained by increased pressure load and ischemic injury during on-pump procedures; indirectly, patients with low PALS generally have more abnormal atrial structure and are more sensitive to perioperative stress^12^. Therefore, our study identifies that patients with low PALS scheduled for on-pump CABG require special attention in POAF prevention efforts^10,13^.

Previous cohort study investigated the relationship between PALS and POAF in patients underwent isolated CABG also showed similar results. Their primary end-point showed that low PALS values had higher risk of developing POAF (HR 3.5, 95% CI: 2.2–5.9, *p* <0.001) and when combined with older age (>70 years) the risk became higher (HR 11.2, 95% CI: 4.7–26.6, *p* <0.001)^13^. Of notes, 95% participants of their study using on-pump CABG surgical technique, hence the impact of inflamatory effect caused by the bypass machine was not explored. Moreover, the follow-up time was only limited to hospital stay. While in our study, surgical technique was varied across the groups and the follow-up time was long and sufficient (30-day after the index CABG) as of POAF might still occur 4 weeks after index CABG^20^.

Other risk factor to predict the occurrence of POAF was POAF score. They described that age, chronic obstructive pulmonary disease, emergency operation, preoperative IABP, left ventricular ejection fraction <30%, estimated glomerular filtration rate <15 mL/min per m^2^ or dialysis, and any heart valve surgery were independent AF predictors^21^. Their study was so different form our study as thay included patients with valve intervention and did not incorporate the value of PALS. Moreover, despite the large numbers of participants, they only monitored POAF during hospital stay.

The mechanism of POAF after cardiac surgery mainly involved three factors: atrial substrate produced by preoperative, surgery induced, and postoperative process. Hypertension, aging, ischemic heart disease and genetic were some of the pre-existing condition that could alter the structural remodelling of left atrium, making it more susceptible to be a substrate for AF^22^. Those pre-existing conditions was known to associate with increase risk of inflammation and oxidative stress, which added more burden to left atrial led to fibrosis, dilatation, and conduction abnormalities^23^. Along with connexin remodelling, electrical remodelling, and inflammatory response resulting from CABG could easily facilitate AF. Extensive areas of fibrosis led to left atrial dysfunction, which can be detected by PALS before any clinical manifestation arose^22^. Previous studies showed that patients who developed POAF had significantly lower PALS values, even with normal left atrial volume and diameter^23,24^. This finding reflected the highly sensitive value of PALS to identify left atrial dysfunction compared to other parameters. Consistently, in our analysis, left atrial volume was not significantly different between the two groups.

Theoretically, our findings are based on the understanding that left atrial strain reflects reservoir, conduit, and contractile functions of the atrium, which are highly influenced by filling pressures and atrial tissue elasticity^25^. Reduced PALS indicates fibrosis, remodelling, or elevated left atrial pressure, all of which predispose to arrhythmias^26^. Moreover, on-pump surgery physiologically causes atrial perfusion disturbances and inflammation, exacerbating the existing arrhythmogenic substrate^27^. Thus, the combination of structural factors (low PALS) and procedural factors (on-pump) logically increases POAF risk pathophysiologically^28^. Accordingly, PALS assessment can be incorporated into preoperative evaluation to identify high-risk patients^29^.

### Clinical implications

The clinical implications of this study are significant for cardiac surgery practice in Indonesia. First, these findings confirm that preoperative PALS assessment can be used as a screening tool to identify patients at high risk of POAF, allowing more targeted preventive interventions. Second, the combination of low PALS and on-pump surgery can inform individualized surgical strategies, such as choosing off-pump techniques for patients with atrial dysfunction. Third, these results may encourage more intensive rhythm monitoring and prophylactic antiarrhythmic therapy for high-risk groups. Fourth, this study provides a basis for developing risk-based perioperative management protocols tailored to the local population.

Moreover, in addition for this finding to be implemented in general population, we need to validate externally and see if this method is applicable and accurate across different population. PALS is very likely to be reproducible in various health centers as this measurements is feasible, practical, non-invasive, and cost effective. Although, a short-course training is needed to make the measurements more reliable and consistent. The current guidelines recommend using beta blocker and amiodarone as a short term prophylaxis to prevent POAF, this applied to all patients undergoing cardiac surgery. By incorporating PALS values and surgical techniques into the guideline, we can identify high risk patients and this group of patients will likely benefit most from the use of this prophylaxis.

## Limitations

This study has several limitations to consider. First, potential confounding factors may not have been fully eliminated, such as variations in perioperative management and post-operative antiarrhythmic drug use. Second, a single-center study with significantly homogeneous gender with a predominance of male gender (91.4%) limits the generalizability of the findings, promotes selection bias, and small sample size. Third, the detection of POAF in our study might be undervalue as we did not use a continuous ECG monitoring or telemetry. This led to potential miss of POAF episode. Further study might consider using a more developed method to overcome this matter. Fourth, other variables potentially influencing POAF occurrence, such as inflammatory status and other biomarkers, were not analyzed in this study.

## Conclusion

This study demonstrates that preoperative PALS <28% is a strong predictor of POAF in patients undergoing isolated CABG. Additionally, subgroup analysis revealed that on-pump CABG technique and the combination of low PALS with on-pump CABG significantly increase POAF risk compared to other groups. Based on these findings, it is expected that PALS assessment can be integrated into preoperative evaluation to identify high-risk patients, enabling more optimal prevention and management of POAF in the CABG patient population moving forward.

## Statements and declarations

### Funding

All funding for this study come from personal funding.

### Author contributions

**Conceptualization and designs:** Ahmad F. Lubis, Andre P. Ketaren, Abdul H. Raynaldo

**Data acquisition:** Ahmad F. Lubis

**Data analysis and interpretation:** Ahmad F. Lubis, Andre P. Ketaren, Abdul H. Raynaldo

**Writing - original draft preparation:** Ahmad F. Lubis

**Writing –Review and editing:** Ahmad F. Lubis, Andre P. Ketaren, Abdul H. Raynaldo, Anggia C. Lubis, Teuku B. Haykal,

**Study Supervision:** Tengku W. Ardini, Joy W. Purba, Nizam Z. Akbar

## Conflict of interest statement

The authors declare that they have no financial conflict of interest with regard to the content of this manuscript.

## Data sharing statement

The datasets generated during and/or analyzed during the current study are not publicly available due to confidential issue about subjects personal information, but are available from the corresponding author on reasonable request.
